# Editorial Notes

**Published:** 1882-11

**Authors:** 


### EDITORIAL NOTES.

WE have received a copy of the Transactions of the Medical Association of the State of Missouri, at its Twenty-Fifth Annual Session, held at Hannibal, May 16, 17 and 18, 1882. It comprises a volume of 220 pages, printed on excellent paper, and shows that our brethren in the West are earnest in their efforts to disseminate their opinions on current medical topics.

THE Beef Peptonoids, an advertisement of which will be found elsewhere in this JOURNAL, have been prominently brought into notice in the treatment of the late President Garfield. Dr. Bliss, in the *New York Medical Record*, of July 15, 1882, furnishes quite an exhaustive article, in which he gives the results of their use in that case. The preparation is worthy of a trial, and we invite the profession to give us the results of their use of these nutritive agents.

THE secular press furnishes as a news-item that Dr. Boynton has presented his account for professional service, rendered to the late President, the amount being $4,500. We are gratified that the position taken by Professors Hamilton and Agnew, in regard to compensation for their consulting attendance, has been adopted by one, at least, of the attending surgeons, and that the amount claimed is within the limits of justice and reason. The exorbitant claim of Dr. Bliss has been a scandal to the profession and a just source of criticism upon the part of the public.

